# Socioeconomic disparities in surgery for carotid artery disease in England

**DOI:** 10.1093/bjsopen/zrad056

**Published:** 2023-07-28

**Authors:** Thaison Tong, Ravi Maheswaran, Jonathan Michaels, Paul Brindley, Stephen Walters, Shah Nawaz

**Affiliations:** School of Health and Related Research, University of Sheffield, Sheffield, South Yorkshire, UK; School of Health and Related Research, University of Sheffield, Sheffield, South Yorkshire, UK; School of Health and Related Research, University of Sheffield, Sheffield, South Yorkshire, UK; Department of Landscape Architecture, University of Sheffield, Sheffield, South Yorkshire, UK; School of Health and Related Research, University of Sheffield, Sheffield, South Yorkshire, UK; Sheffield Vascular Institute, Sheffield Teaching Hospitals NHS Foundation Trust, Sheffield, South Yorkshire, UK

## Abstract

**Background:**

Carotid artery disease and stroke are more prevalent in socioeconomically deprived areas. The aim was to investigate socioeconomic disparities in carotid artery disease surgery rates and in outcomes following surgery.

**Methods:**

The study used population-based ecological and cohort study designs, 31 672 census areas in England, hospital admissions from April 2006 to March 2018, the Index of Multiple Deprivation 2010 as the area-level deprivation indicator, and Poisson, logistic, and Cox regression.

**Results:**

A total of 54 377 patients (67 per cent men) from a population aged 55 years and older of 14.7 million had carotid artery disease procedures (95 per cent carotid endarterectomy). Carotid endarterectomy rates were 116 per cent (95% c.i. 101 to 132) higher in men and 180 per cent (95% c.i. 155 to 207) higher in women aged 55–64 years in the most compared with the least socioeconomically deprived areas by quintile. However, this difference diminished and appeared to reverse with increasing age, with 24 per cent (95% c.i. 14 to 33) and 12 per cent (95% c.i. −3 to 24) lower carotid endarterectomy rates respectively in men and women aged 85 years and older in the most deprived areas. Patients in deprived areas having carotid endarterectomy were more likely to have been admitted as symptomatic emergency carotid artery disease admissions. Mortality, and a combined outcome of mortality or stroke-related re-admission, were both worse in patients living in more deprived areas and were only partially accounted for by the higher prevalence of co-morbidities. There was, however, no clear pattern of association between deprivation and elective waiting time for carotid endarterectomy.

**Conclusions:**

These results provide evidence of socioeconomic disparities in surgery for carotid artery disease. Clear policies are needed to address these disparities.

## Teaser

This nationwide population-based study of surgery for carotid artery disease in England, which has a National Health Service, examined data from 2006 to 2018. It found that patients having carotid endarterectomy who were living in more socioeconomically deprived areas were more likely to have presented with symptomatic carotid artery disease and have worse survival following surgery.

Whilst population-based surgery rates were higher in more deprived areas in the middle-aged population, this diminished and reversed with increasing age, with lower rates in more deprived areas in the population aged 85+ years. Clear policies and guidelines are needed to address these disparities.

## Introduction

Stroke is a major cause of mortality and morbidity worldwide^1,2^. Carotid artery disease (CAD) may be responsible for about 10–20 per cent of strokes^3^, and there are clear socioeconomic gradients in both stroke risk and the prevalence of CAD, with higher rates in more socioeconomically disadvantaged sections of the population^4–7^.

Options for the management of carotid artery stenosis include surgical interventions^3^. For example, in the UK, national guidelines recommend surgical intervention for symptomatic patients with severe carotid artery stenosis^8^. Carotid endarterectomy (CEA) is the preferred option, with carotid artery stenting (CAS) reserved for patients who are unsuitable for CEA^9^.

Whilst there is a clear socioeconomic gradient in CAD^6,7^, very few studies have investigated whether there are socioeconomic disparities in population-based surgery rates for CAD^10–12^. The terms CAD surgery and CAD procedure are used to include both CEA and CAS. Given the higher prevalence of CAD in more socioeconomically disadvantaged populations, a higher CAD surgery rate might be expected in more deprived areas. However, the evidence is inconsistent. Studies in England and Germany found higher rates in more deprived areas^10,11^, but a study in Scotland found no evidence of association^12^.

In addition, relatively few studies have examined socioeconomic disparities in outcomes following surgery for CAD^13–18^. Most were carried out in the USA but include one in Italy^18^. The range of outcomes examined included mortality, stroke, and re-admissions following surgery. The limited evidence is inconclusive, with some studies finding worse outcomes in patients from disadvantaged backgrounds^13,14^, but others finding no evidence of socioeconomic disparities^15–18^.

Access to healthcare is substantially influenced by healthcare funding systems^19^. In England, the National Health Service (NHS) provides universal access to healthcare that is centrally funded mainly through general taxation. It is largely free at the point of delivery and there is no charge for CAD surgery. The aim of this national population-based study was to investigate socioeconomic disparities in surgery rates for CAD and in outcomes following surgery.

## Methods

### Hospital admissions and linked mortality data

Hospital Episode Statistics (HES) data on admissions to NHS hospitals in England from 1 April 2006 to 31 March 2018, with linked mortality records, were obtained from NHS Digital, the national agency which manages all NHS hospital data in England. All admissions for CAD procedures were identified using OPCS procedure codes, the standard classification system used in the English NHS^20^, and individual patients with a CAD procedure were identified using a pseudoanonymised patient identifier.

For each patient, the index admission was defined as the first admission with a CAD procedure and the analysis was carried out as a patient-based analysis. Patients were then classified into four categories based on whether their index admission in HES was recorded as an elective or an emergency admission. An elective admission is a planned admission that has been arranged in advance; an emergency admission is an unplanned, unpredictable admission at short notice because of clinical need. The four categories are: elective CEA; elective CAS; emergency CEA; and emergency CAS. Information on co-morbidities was derived from the International Classification of Diseases 10th Revision (ICD-10) codes recorded in the diagnosis fields for each admission record. Seven categories of co-morbidities were considered (coronary artery disease, heart failure, chronic obstructive pulmonary disease (COPD), diabetes, renal disease, cancer, moderate/severe liver disease). These were based on the Royal College of Surgeons Charlson Score and opinions from the expert clinical advisory panel for this project^21,22^. Ethnicity of patients was extracted from the most recent admission record. The study was approved by the University of Sheffield Research Ethics Committee (Ref: 037926).

### Geography, population data, and socioeconomic deprivation

Each patient in the HES data set had been assigned to a lower layer super output area (LSOA) of residence. These are census areas with an average population of 1500 people^23^. The 2011 Census changed a small number of LSOAs compared with the 2001 Census. The analysis was restricted to 31 672 of the 32 844 LSOAs in 2011 (96.4 per cent) to maintain consistency across the study time span.

Mid-year population estimates by age and sex, available at the LSOA level, were used to calculate CAD procedure rates. The Income Domain from the Index of Multiple Deprivation (IMD) 2010 was used as the indicator of socioeconomic deprivation at the LSOA level (a neighbourhood area-level indicator)^24^. The IMD is the national index of deprivation widely used by government agencies in England.

### Study design and statistical analysis

A population-based (ecological) study design was used to examine CAD procedure rates and a cohort study design was used to examine survival patterns after surgery. Postoperative survival patterns were investigated in terms of both overall survival and a composite of overall or stroke re-admission-free survival. For overall survival, the endpoint was date of death. For the composite outcome, the endpoint was either date of subsequent stroke-related admission or date of death. Stroke-related admission was defined as the first admission after the index admission with a primary diagnosis of stroke. Follow-up was to 31 March 2018 and analyses were restricted to patients aged 55 years and older.

The percentage of patients having CAD surgery who had been admitted with symptomatic CAD was also investigated. A pragmatic approach was used to define symptomatic CAD. This included all patients having CAD surgery in an emergency admission but also included patients having elective CAD surgery who had an emergency admission within the previous 90 days for symptomatic CAD (that is admitted with a primary diagnosis of stroke, transient ischaemic attack, or stenosis of the carotid artery).

Poisson, logistic, and Cox regression were used as appropriate to examine associations between socioeconomic deprivation and procedure rates, admission patterns, and survival following operation. Variables included as appropriate to adjust for confounding included age, sex, year of admission, ethnicity, co-morbidities, presence of stroke in the index admission, and previous stroke-related admission (within 90 days preceding the index admission). Ethnicity categories were collapsed into three groups for the adjustment in regression analyses: White, non-White, and Missing.

Because data were very sparse at the LSOA level, LSOAs were grouped into five categories using deprivation quintiles. The median deprivation value within each category was used as a continuous variable in the statistical analyses. Rate ratios, odds ratios, and hazard ratios were calculated as a trend across all categories and presented as the ratio, with 95 per cent c.i., for the most relative to the least deprived category.

## Results

### Characteristics of patients

There were 54 377 patients aged 55 years and older who had CAD procedures in NHS hospitals in England over the 12-year study period, with a corresponding average denominator population of 14.7 million. Sixty-seven per cent of the patients were men and 7 per cent were 85 years and older (*Table 1*). The majority had elective CEA (66 per cent), followed by emergency CEA (29 per cent), elective CAS (3 per cent), and emergency CAS (2 per cent). Of the seven co-morbidities examined, coronary heart disease (24 per cent of patients), diabetes (23 per cent of patients), and COPD (19 per cent of patients) were the most common. Some 93.8 per cent of patients were White, 3.9 per cent were non-White, and 2.3 per cent did not have ethnicity information recorded (*Table 1*).

**Table 1:** Characteristics of patients receiving surgery for carotid artery disease in England (April 2006 to March 2018)

Characteristic	Men	Women	All
	*n* (%)	*n* (%)	*n* (%)
**Age (years)**			
55–64	7258 (20)	3373 (19)	10 631 (20)
65–74	14 288 (39)	6474 (36)	20 762 (38)
75–84	12 522 (34)	6501 (36)	19 023 (35)
85 and older	2381 (7)	1580 (9)	3961 (7)
**Procedure**)			
Elective CAS	1038 (3)	576 (3)	1614 (3)
Elective CEA	24 452 (67)	11 595 (65)	36 047 (66)
Emergency CAS	580 (2)	336 (2)	916 (2)
Emergency CEA	10 379 (28)	5421 (30)	15 800 (29)
**Co-morbidities**)			
Coronary artery disease	9567 (26)	3622 (20)	13 189 (24)
Heart failure	1978 (5)	886 (5)	2864 (5)
COPD	6417 (18)	3854 (21)	10 271 (19)
Diabetes	8664 (24)	3950 (22)	12 614 (23)
Renal disease	2154 (6)	1046 (6)	3200 (6)
Cancer	2545 (7.0)	722 (4.0)	3267 (6.0)
Moderate/severe liver disease	76 (0.2)	25 (0.1)	101 (0.2)
**Ethnicity**)			
White	34 080 (93.5)	16 909 (94.3)	50 989 (93.8)
South Asian*	871 (2.4)	326 (1.8)	1197 (2.2)
Black	176 (0.5)	92 (0.5)	268 (0.5)
Mixed	58 (0.2)	22 (0.1)	80 (0.1)
Others	433 (1.2)	184 (1.0)	617 (1.1)
Missing	831 (2.3)	395 (2.2)	1226 (2.3)
All	36 449 (100)	17 928 (100)	54 377 (100)

Data are *n* (%). *Indian, Pakistani, or Bangladeshi. CAS, carotid artery stenting; CEA, carotid endarterectomy; COPD, chronic obstructive pulmonary disease.

General trends over time in population-based rates, and general patterns in survival curves following surgery for CAD, are presented in the *Supplementary Material*. Elective CEA rates decreased over time while emergency CEA rates showed an inconsistent increase (*Fig. S1*). The worst postoperative survival pattern was seen following emergency CAS (*Fig. S2*).

As 95 per cent of CAD procedures were by CEA, this was the focus of the analysis in relation to socioeconomic deprivation.

### Socioeconomic deprivation and population-based carotid endarterectomy rates

*Table 2* presents population-based CEA rates by deprivation category, sex, and age group. It also presents ratios of rates in the most, relative to the least, socioeconomically deprived areas, adjusted for year of admission.

**Table 2:** Annual average population rates by deprivation category, and rate ratios (adjusted for year of admission) for CEA in the most relative to the least socioeconomically deprived category by age and sex; England (April 2006 to March 2018)

Age (years)	*n*	CEA rate per 100 000 population (*n*)					Rate ratio (95% c.i.)
		Dep 1 (most deprived)	Dep 2	Dep 3	Dep 4	Dep 5 (least deprived)	
**Men**							
55–64	6848	29.6 (1679)	23.3 (1500)	18.7 (1357)	16.8 (1328)	12.3 (984)	2.16 (2.01, 2.32)
65–74	13 607	64.5 (2522)	58.9 (2796)	50.6 (2881)	45.7 (2894)	40.8 (2514)	1.54 (1.46, 1.62)
75–84	12 092	81.7 (1811)	81.9 (2265)	79.3 (2611)	77.5 (2771)	76.1 (2634)	1.07 (1.03, 1.12)
85 and older	2284	42.6 (272)	45.5 (403)	44.3 (461)	54.2 (592)	54.7 (556)	0.76 (0.67, 0.86)
**Women**							
55–64	3088	15 (865)	10.2 (677)	8.3 (628)	6.5 (533)	4.7 (385)	2.80 (2.55, 3.07)
65–74	6117	30.8 (1321)	24.2 (1269)	21.3 (1322)	17.6 (1198)	15.3 (1007)	1.89 (1.75, 2.04)
75–84	6275	35.1 (1079)	33.1 (1265)	33.2 (1430)	29.8 (1321)	28.7 (1180)	1.20 (1.13, 1.28)
85 and older	1536	15.8 (222)	15.2 (293)	16.2 (349)	16.2 (346)	18.3 (326)	0.88 (0.76, 1.03)

CEA, carotid endarterectomy; Dep, deprivation category by quintile.

CEA rates were substantially higher in the most relative to the least deprived areas in the 55–64 years age group; the rate was 116 per cent (95% c.i. 101 to 132) higher in men and 180 per cent (95% c.i. 155 to 207) higher in women in this age group. However, the magnitude of the rate ratios diminished with increasing age and reversed in the over 85 years age group, with CEA rates 24 per cent (95% c.i. 14 to 33) lower in men and 12 per cent (95% c.i. −3 to 24) lower in women in the most deprived compared with the least deprived areas.

### Socioeconomic deprivation, co-morbidities, and symptomatic emergency admissions

*Table 3* presents the percentage of CEA patients with co-morbidities by deprivation category, and adjusted odds ratios comparing the proportions in the most relative to the least deprived category. The odds of having co-morbidities were higher in the most deprived areas for most co-morbidities, especially for COPD where patients from the most deprived areas had 91 per cent (95% c.i. 79 to 103) higher odds of COPD compared with patients from the least deprived areas.

**Table 3:** Co-morbidities by deprivation category in patients admitted for CEA in England (April 2006 to March 2018)

Co-morbidity	Percentage with co-morbidity (*n*)					Odds ratio*(95% c.i.)
	Dep 1 (most deprived)	Dep 2	Dep 3	Dep 4	Dep 5 (least deprived)	
Coronary artery disease	26.6% (2597)	25.2% (2641)	23.6% (2608)	22.3% (2449)	22.3% (2139)	1.34 (1.26, 1.42)
Heart failure	5.9% (580)	5.3% (553)	5.1% (562)	4.9% (534)	4.7% (452)	1.36 (1.22, 1.52)
COPD	24.6% (2403)	20.6% (2161)	18.2% (2014)	15.7% (1725)	15% (1434)	1.92 (1.81, 2.04)
Diabetes	26.9% (2627)	24.4% (2551)	23.1% (2546)	20.9% (2295)	20.9% (2007)	1.33 (1.26, 1.41)
Renal disease	6.4% (628)	5.7% (598)	5.7% (632)	5.6% (619)	5.8% (552)	1.32 (1.19, 1.47)
Cancer	5.8% (562)	5.7% (594)	5.5% (605)	6.2% (685)	6.1% (580)	1.04 (0.94, 1.16)
Moderate/severe liver disease	0.2% (22)	0.2% (23)	0.2% (18)	0.1% (12)	0.2% (19)	1.32 (0.76, 2.32)
Total no. of CEA patients	9771	10 468	11 039	10 983	9586	

*Odds ratios calculated as a trend across all categories, adjusted for age, sex, year, and ethnicity, and expressed as the ratio for the most relative to the least deprived category. CEA, carotid endarterectomy; Dep, deprivation category by quintile; COPD, chronic obstructive pulmonary disease.

*Table 4* presents the percentage of CEA patients admitted as a symptomatic emergency with CAD by deprivation category, and odds ratios comparing the proportions in the most relative to the least deprived categories. Patients having CEA who were living in deprived areas were more likely to have been admitted as symptomatic emergency CAD admissions, with those living in the most deprived areas having 25 per cent (95% c.i. 19 to 31) higher odds of symptomatic emergency admission compared with patients living in the least deprived areas. Adjustment for co-morbidities made little difference to the odds ratios.

**Table 4:** Percentage of patients having CEA who had been admitted as a symptomatic emergency with CAD, by socioeconomic deprivation category, and adjusted odds ratios for the most relative to the least deprived category; England (April 2006 to March 2018)

Age (years)	% with symptomatic admission (*n*/N)					Model 1 Odds ratio* (95% c.i.)	Model 2 Odds ratio* (95% c.i.)
	Dep 1 (most deprived)	Dep 2	Dep 3	Dep 4	Dep 5 (least deprived)		
**Men**							
55–64	46.2 (776/1679)	47.5 (712/1500)	45.3 (615/1357)	44.7 (593/1328)	42.4 (417/984)	1.09 (0.96, 1.25)	1.11 (0.97, 1.27)
65–74	49.7 (1254/2522)	46.9 (1312/2796)	45.4 (1307/2881)	45.0 (1301/2894)	43.9 (1103/2514)	1.32 (1.20, 1.46)	1.31 (1.18, 1.44)
75–84	57.0 (1033/1811)	52.9 (1199/2265)	52.1 (1360/2611)	50.9 (1410/2771)	50.8 (1337/2634)	1.34 (1.20, 1.50)	1.32 (1.18, 1.48)
85+	65.8 (179/272)	65.0 (262/403)	67.0 (309/461)	64.0 (379/592)	60.4 (336/556)	1.28 (0.97, 1.71)	1.28 (0.96, 1.7)
All men	51.6 (3242/6284)	50.0 (3485/6964)	49.1 (3591/7310)	48.6 (3683/7585)	47.7 (3193/6688)	1.27 (1.20, 1.36)	1.26 (1.19, 1.35)
**Women**							
55–64	45.9 (397/865)	45.6 (309/677)	40.0 (251/628)	43.9 (234/533)	41.3 (159/385)	1.15 (0.95, 1.40)	1.14 (0.94, 1.40)
65–74	47.4 (626/1321)	45.3 (575/1269)	44.3 (586/1322)	45.8 (549/1198)	44.6 (449/1007)	1.13 (0.98, 1.31)	1.10 (0.95, 1.27)
75–84	57.6 (622/1079)	55.8 (706/1265)	53.9 (771/1430)	53.1 (702/1321)	51.8 (611/1180)	1.31 (1.13, 1.53)	1.28 (1.10, 1.49)
85+	64.0 (142/222)	66.6 (195/293)	65.9 (230/349)	65.0 (225/346)	63.8 (208/326)	1.15 (0.82, 1.60)	1.15 (0.83, 1.61)
All women	51.2 (1787/3487)	50.9 (1785/3504)	49.3 (1838/3729)	50.3 (1710/3398)	49.2 (1427/2898)	1.20 (1.10, 1.31)	1.17 (1.07, 1.28)
**All**	51.5 (5029/9771)	50.3 (5270/10 468)	49.2 (5429/11 039)	49.1 (5393/10 983)	48.2 (4620/9586)	1.25 (1.19, 1.31)	1.23 (1.17, 1.30)

*Odds ratios calculated as a trend across all categories and expressed as the ratio for the most relative to the least deprived category. Model 1: adjusted for year, age, sex, and ethnicity. Model 2: adjusted for year, age, sex, ethnicity, and co-morbidities. (*n*/N), *n* is the number of symptomatic CEA patients and N is the total number of CEA patients. CEA, carotid endarterectomy; Dep, deprivation category by quintile.

### Socioeconomic deprivation and survival following carotid endarterectomy

Mortality following CEA was 42 per cent (95% c.i. 36 to 48) higher in the most relative to the least deprived areas (*Table 5*). Adjustment for co-morbidities, including a stroke diagnosis in a previous admission or in the index admission, reduced the excess hazard to 29 per cent (5% c.i. 18 to 40) suggesting that some of the excess mortality was accounted for by co-morbidities. Similar patterns were observed for the composite outcome of mortality or stroke-related re-admission.

**Table 5:** Adjusted hazard ratios* for mortality, and mortality or stroke-related re-admission, following CEA, in the most relative to the least socioeconomically deprived category by age and sex; England (April 2006 to March 2018)

Mortality after CEA					
	Age (years)	*n*	Deaths	Model 1 Hazard ratio* (c.i.)	Model 2 Hazard ratio* (c.i.)
Men	55–64	6848	1213	1.62 (1.39, 1.88)	1.52 (1.17, 1.98)
	65–74	13 605	3813	1.65 (1.51, 1.80)	1.53 (1.33, 1.77)
	75–84	12 087	5203	1.28 (1.18, 1.38)	1.07 (0.94, 1.22)
	85+	2284	1292	1.34 (1.13, 1.60)	1.42 (1.05, 1.91)
	All	34 824	11 521	1.44 (1.37, 1.52)	1.29 (1.18, 1.40)
Women	55–64	3088	463	1.69 (1.32, 2.15)	1.05 (0.69, 1.61)
	65–74	6114	1597	1.52 (1.33, 1.73)	1.34 (1.07, 1.68)
	75–84	6275	2528	1.24 (1.11, 1.39)	1.16 (0.96, 1.40)
	85+	1536	822	1.26 (1.02, 1.55)	1.03 (0.69, 1.52)
	All	17 013	5410	1.36 (1.26, 1.47)	1.20 (1.06, 1.37)
All	All	51 837	16 931	1.42 (1.36, 1.48)	1.26 (1.17, 1.35)
**Mortality or stroke-related re-admission after CEA**					
	**Age (years)**	***n***	**Death or re-admission**	**Model 1** Hazard ratio* (c.i.)	**Model 2** Hazard ratio* (c.i.)
Men	55–64	6848	1482	1.63 (1.43, 1.87)	1.53 (1.21, 1.94)
	65–74	13 605	4320	1.61 (1.48, 1.75)	1.51 (1.32, 1.73)
	75–84	12 087	5571	1.26 (1.17, 1.36)	1.09 (0.96, 1.23)
	85+	2284	1337	1.32 (1.11, 1.56)	1.39 (1.03, 1.88)
	All	34 824	12 710	1.43 (1.36, 1.5)	1.29 (1.19, 1.40)
Women	55–64	3088	565	1.81 (1.46, 2.26)	1.13 (0.78, 1.64)
	65–74	6114	1782	1.55 (1.37, 1.76)	1.42 (1.15, 1.77)
	75–84	6275	2735	1.19 (1.07, 1.33)	1.10 (0.92, 1.32)
	85+	1536	868	1.22 (1.00, 1.50)	1.03 (0.70, 1.52)
	All	17 013	5950	1.36 (1.27, 1.46)	1.21 (1.07, 1.37)
All	All	51 837	18 660	1.41 (1.35, 1.46)	1.26 (1.18, 1.34)

*Hazard ratios calculated as a trend across all deprivation (quintile) categories and expressed as the ratio for the most relative to the least deprived category. Model 1: adjusted for year, age, sex, emergency admission, and ethnicity. Model 2: adjusted for year, age, sex, emergency admission, ethnicity, and co-morbidities. CEA, carotid endarterectomy.

### Changes in elective waiting time for carotid endarterectomy and socioeconomic deprivation

**Fig. 1:**
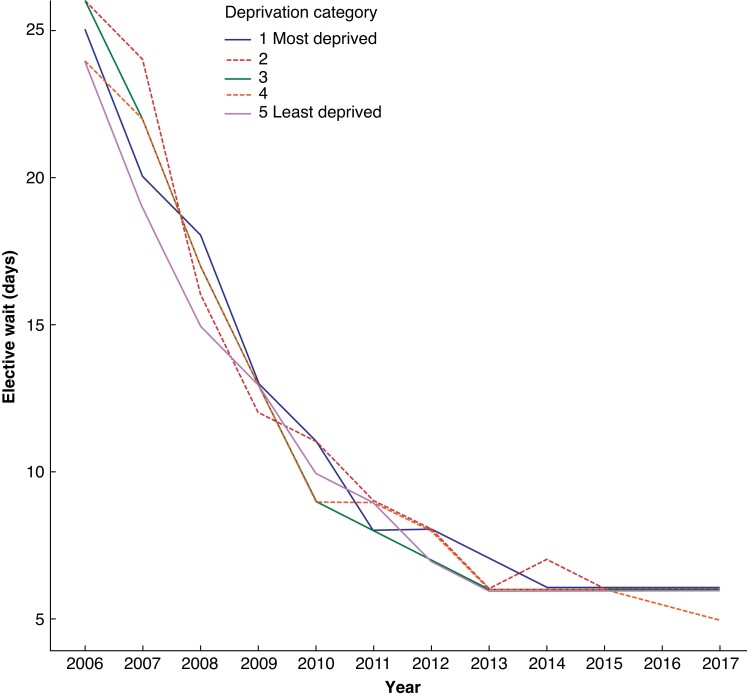
Median waiting time for elective carotid endarterectomy, by year and deprivation category; England (April 2006 to March 2018)

National guidance regarding CAD management, including urgency of treatment, was issued during the time period examined in this study in response to emerging evidence^25,26^. This resulted in a steep decline in elective waiting time from 2006 (median of 25 days) to 2013 (median of 6 days), followed by a plateau (*Fig. 1*). There was, however, no clear pattern of association between socioeconomic deprivation and elective waiting time.

## Discussion

Population-based CEA rates were higher in more socioeconomically deprived areas in the population aged 55–64 years. However, this diminished with increasing age and was, in fact, lower in more deprived areas in the population aged 85 years and older. Patients having CEA who were living in more deprived areas were more likely to have presented with symptomatic CAD. Survival following CEA was worse in patients living in more deprived areas and was only partially accounted for by the higher prevalence of co-morbidities in patients from these areas. There was, however, no clear pattern of association between deprivation and waiting times for elective CEA.

Higher CEA rates in more socioeconomically deprived areas might be expected because the need may be greater, as indicated by higher rates of stroke and CAD in more disadvantaged populations^4–7^. A national study in Germany based on 88 182 CAD procedures found higher surgery rates in more deprived areas^11^. A small study in two regions in the north of England based on 1232 operations found that the CEA rate was lower in the least deprived area by quintile but with no evidence of a trend across the other deprivation categories^10^. A small study in a part of Scotland, based on only 192 CEAs, found no apparent association between CEA rates and deprivation^12^.

In contrast, this study found that whilst there was clear evidence of higher CEA rates in more deprived areas in the 55–64 years age group, the magnitude diminished with increasing age and, in fact, reversed in the 85 years and older age group, resulting in lower rates in this age group in more deprived areas. This may indicate socioeconomic disparity in access to CEA amongst older people living in more deprived areas. A potential alternative explanation is that the underlying higher prevalence of carotid stenosis in more deprived areas diminishes with increasing age, which would be consistent with the observed pattern. There is some evidence suggesting that higher stroke rates in deprived relative to affluent areas diminishes with increasing age and reverses in the oldest age groups^27–29^. This phenomenon of reversal has also been observed in relation to surgery rates for abdominal aortic aneurysm^30^. However, there are no robust data on the prevalence of carotid stenosis by both age and socioeconomic deprivation.

Relatively few studies have examined if there are socioeconomic disparities in outcomes following surgery for CAD^13–18^, and most were carried out in the USA^13–17^. Vogel *et al.* examined data on 234 825 patients who had surgery for CAD^13^. They used the median household income in ZIP codes (areas) as the proxy for socioeconomic deprivation and found that higher levels of deprivation were associated with increased in-hospital mortality and postoperative stroke. Hicks *et al.* analysed data on 51 942 patients who had surgery for CAD, used payer (insurance) status as a proxy for deprivation, and found that high-risk patients in the deprived category had an increased risk of stroke within a 2-year follow-up period^14^.

However, Schneider *et al.* analysed data on 394 835 CAD procedures and found no evidence of association between payer status and either in-hospital mortality or postoperative stroke^15^. Panchap *et al.* analysed data on 198 120 admissions for CAD procedures and used payer status and income as measures of deprivation^17^. They also found no association between in-hospital mortality or postoperative stroke and either measure of deprivation. In an Italian study of in-hospital outcomes following CEA in four cities, Agabiti *et al.* analysed data on 5427 admissions^18^. They too found no evidence of association between an area-level (census-based) indicator of deprivation and in-hospital mortality after CEA.

In contrast, this study found clear evidence of worse survival following CEA in patients living in more deprived areas, and also clear evidence of worse outcomes when a composite measure of mortality or stroke following CEA was used. The worse outcomes were only partially accounted for by the higher prevalence of co-morbidities in patients from deprived areas. A key factor which distinguishes this study from the previous ones is that survival and stroke risk following CEA were assessed over a considerably longer follow-up period of up to 12 years. The three studies finding no evidence of association only examined in-hospital outcomes^15,17,18^, whereas the study by Hicks *et al.* which found an association with stroke risk had a 2-year follow-up period^14^. Another key distinguishing feature is that this study used an area-based index of socioeconomic deprivation available on a continuous scale which allowed for a finer gradation of deprivation. It is interesting to note that Vogel *et al.,* who also used an area-based measure, found an association between deprivation and in-hospital mortality and stroke^13^.

Whilst the focus of the analysis was on CEA, the survival curves showed that the worst survival was in patients who had emergency CAS. A possible explanation is patient selection where the procedure was mainly carried out on high-risk patients who would have been unsuitable for CEA.

Even in a health system where surgery for CAD is free at the point of use, there were socioeconomic disparities, and there may be complex potential explanations for these disparities. The observation that patients having CEA who were living in more deprived areas were more likely to have presented with symptomatic CAD suggests that consideration of treatment for asymptomatic patients may have been more common in less deprived areas. Access to healthcare, in general, may be worse in disadvantaged areas despite higher levels of health need, a situation that has been described as the Inverse Care Law^31^. Socioeconomic deprivation may influence general practitioners’ referral decisions and navigation of the healthcare system^32^.

Literature reviews have found that there is a social gradient in doctor–patient communication, with patients from higher social classes able to communicate more actively, elicit more information, and partake more effectively in shared decision-making^33,34^. In addition, the greater need of patients with multimorbidity in deprived areas has not been reflected in longer consultation length or higher patient-centred care, with higher empathy from general practitioners found in more affluent areas^35^. A systematic review found evidence of implicit (unconscious) bias in healthcare professionals, with higher levels of implicit bias associated with lower quality of care^36^.

The key strengths of this study are that it is one of the largest to date examining population-based surgery rates for CAD in relation to deprivation and that it has the longest length of postoperative follow-up in relation to deprivation using a population-based cohort. Nevertheless, there are a number of limitations which need to be taken into account when interpreting the results. This study did not investigate primary healthcare factors that could have influenced local access to surgery for CAD. Classifying admissions as elective or emergency admissions was based upon HES classification of admission method, which may have been affected by local policies regarding admission for urgent surgery. The study did not have data on patients who were not operated on and could therefore not investigate if turndown rates correlated with socioeconomic deprivation. There may have been errors in diagnostic and operative procedure coding and coding practices may have changed over time. A re-admission with a recorded primary diagnosis of stroke may have identified some patients admitted for another reason, for example a fall, whose previous stroke was coded again.

There was limited information on co-morbidities, which could have resulted in residual confounding despite adjustment using the available information on co-morbidities. Whilst the ethnicity data were used to adjust for confounding by ethnicity, the very small numbers of patients from minority ethnic groups meant that analysis by ethnicity would have lacked power to reliably detect ethnic disparities. The HES data set is primarily an administrative data set and has limited clinical information. The analysis used a small area level socioeconomic indicator which was effective in detecting area-level deprivation effects. However, the level of deprivation experienced by people living in a deprived area might vary, for example by age and sex. In addition, the HES data set did not include an indicator of deprivation at the individual level, which meant that additional assessment of deprivation effects at the individual level could not be assessed.

Socioeconomic disparities in surgery rates and outcomes are not unique to CAD and have been identified in relation to other types of surgery. However, whilst there is a body of literature on causes of surgical disparities, there is very limited evidence on evaluation of the effectiveness of interventions to reduce disparities in surgical healthcare^37^.

Possible approaches to address disparities in CAD surgery rates and outcomes in socioeconomically disadvantaged areas include improving pathways and processes for identifying patients with symptomatic CAD early, rapid investigation of these patients to identify those who would benefit from CEA, and arranging prompt surgery. In addition, medical treatment for CAD and co-morbidities could be optimized to reduce surgery risks and improve postoperative survival and stroke risk.

## Supplementary Material

zrad056_Supplementary_Data

## Data Availability

The HES data used in this project may be obtained from NHS Digital.

## References

[1] Donnan GA , Fisher M, Macleod M, Davis SM. Stroke. Lancet 2008;371:1612–1623 18468545 10.1016/S0140-6736(08)60694-7

[2] GBD 2016 Causes of Death Collaborators . Global, regional, and national age-sex specific mortality for 264 causes of death, 1980–2016: a systematic analysis for the Global Burden of Disease Study 2016. Lancet 2017;390:1151–1210 28919116 10.1016/S0140-6736(17)32152-9 PMC5605883

[3] Mortimer R , Nachiappan S, Howlett D. Carotid artery stenosis screening: where are we now? Br J Radiol 2018;91:20170380 10.1259/bjr.20170380 PMC6350500 29770736

[4] Jakovljević D , Sarti C, Sivenius J, Torppa J, Mähönen M, Immonen-Räihä P et al Socioeconomic status and ischemic stroke: the FINMONICA stroke register. Stroke 2001;32:1492–1498 11441191 10.1161/01.str.32.7.1492

[5] Akyea R , Vinogradova Y, Qureshi N, Patel R, Kontopantelis E, Ntaios G et al Sex, age, and socioeconomic differences in nonfatal stroke incidence and subsequent major adverse outcomes. Stroke 2021;52:396–405 33493066 10.1161/STROKEAHA.120.031659 PMC7834661

[6] Lynch J , Kaplan G, Salonen R, Cohen R, Salonen J. Socioeconomic status and carotid atherosclerosis. Circulation 1995;92:1786–1792 7671362 10.1161/01.cir.92.7.1786

[7] Nash SD , Cruickshanks KJ, Klein R, Klein BE, Nieto FJ, Ryff CD et al Socioeconomic status and subclinical atherosclerosis in older adults. Prev Med 2011;52:208–212 21195728 10.1016/j.ypmed.2010.12.009 PMC3062713

[8] NICE . Stroke and transient ischaemic attack in over 16s: diagnosis and initial management. Published 2019. Accessed 15 Feb 2022. Available from: https://www.nice.org.uk/guidance/ng128 31211538

[9] Waton S , Johal A, Birmpili P, Li Q, Cromwell D, Pherwani A et al National Vascular Registry: 2020 Annual Report. Published 2020. Accessed 15 Feb 2022. Available from: https://www.vsqip.org.uk/reports/2020-annual-report/

[10] Oliver SE , Thomson RG. Are variations in the use of carotid endarterectomy explained by population need? A study of health service utilisation in two English health regions. Eur J Vasc Endovasc Surg 1999;17:501–506 10375486 10.1053/ejvs.1999.0792

[11] Kuehnl A , Salvermoser M, Knipfer E, Zimmermann A, Schmid V, Eckstein HH. Regional frequency variation of revascularization procedures for carotid stenosis in Germany: secondary data analysis of DRG data from 2012 to 2014. Gefasschirurgie 2018;23:56–65 30147245 10.1007/s00772-018-0415-7 PMC6096714

[12] MacKenzie R , Nimmo F, Bachoo P, Alozairi O, Brittenden J. The relationship between socio-economic status, geography, symptomatic carotid territory disease and carotid endarterectomy. Eur J Vasc Endovasc Surg 2003;26:145–149 12917828 10.1053/ejvs.2002.1949

[13] Vogel TR , Kruse RL, Kim RJ, Dombrovskiy VY. Racial and socioeconomic disparities after carotid procedures. Vasc Endovascular Surg 2018;52:330–334 29554858 10.1177/1538574418764063

[14] Hicks CW , Nejim B, Locham S, Aridi HD, Schermerhorn ML, Malas MB. Association between Medicare high-risk criteria and outcomes after carotid revascularization procedures. J Vasc Surg 2018;67:1752–1761.e2 29361324 10.1016/j.jvs.2017.10.066

[15] Schneider EB , Black JH, Hambridge HL, Lum YW, Freischlag JA, Perler BA et al The impact of race and ethnicity on the outcome of carotid interventions in the United States. J Surg Res 2012;177:172–177 22459294 10.1016/j.jss.2012.02.050

[16] Hintze AJ , Greenleaf EK, Schilling AL, Hollenbeak CS. Thirty-day readmission rates for carotid endarterectomy versus carotid artery stenting. J Surg Res 2019;235:270–279 30691806 10.1016/j.jss.2018.10.011

[17] Panchap L , Safavynia SA, Tangel V, White RS. Socioeconomic disparities in carotid revascularization procedures. J Cardiothorac Vasc Anesth 2019;pii:S1053-0770(19)31210-8 10.1053/j.jvca.2019.11.038 31917077

[18] Agabiti N , Cesaroni G, Picciotto S, Bisanti L, Caranci N, Costa G et al The association of socioeconomic disadvantage with postoperative complications after major elective cardiovascular surgery. J Epidemiol Community Health 2008;62:882–889 18791046 10.1136/jech.2007.067470 PMC2602741

[19] Besley T , Gouveia M. Alternative systems of health care provision. Economic Policy 1994;9:199–258

[20] NHS Digital . OPCS Classification of Interventions and Procedures. 2019 [updated 4 December 2019]. Available from: https://digital.nhs.uk/data-and-information/information-standards/information-standards-and-data-collections-including-extractions/publications-and-notifications/standards-and-collections/dcb0084-opcs-classification-of-interventions-and-procedures

[21] Armitage JN , van der Meulen JH. Identifying co-morbidity in surgical patients using administrative data with the Royal College of Surgeons Charlson score. Br J Surg 2010;97:772–781 20306528 10.1002/bjs.6930

[22] Michaels J , Wilson E, Maheswaran R, Radley S, Jones G, Tong TS et al Configuration of vascular services: a multiple methods research programme. NIHR Journals Library. 2021; Programme Grants for Applied Research. 10.3310/pgfar09050 33900707

[23] ONS . Census geography—An overview of the various geographies used in the production of statistics collected via the UK census. Published 2021. Accessed 10 Jan 2022. Available from: https://www.ons.gov.uk/methodology/geography/ukgeographies/censusgeography

[24] Communities and Local Government . The English Indices of Deprivation 2010. Published 2011. Accessed 15 Feb 2022. Available from: https://assets.publishing.service.gov.uk/government/uploads/system/uploads/attachment_data/file/6871/1871208.pdf

[25] Rothwell P , Giles M, Chandratheva A, Marquardt L, Geraghty O, Redgrave J et al Effect of urgent treatment of transient ischaemic attack and minor stroke on early recurrent stroke (EXPRESS study): a prospective population-based sequential comparison. Lancet 2007;370:1432–1442 17928046 10.1016/S0140-6736(07)61448-2

[26] The Vascular Society of Great Britain and Ireland . The provision of services for patients with vascular disease. Published 2015. Accessed 10 Jan 2022. Available from: https://www.vascularsociety.org.uk/_userfiles/pages/files/Resources/POVS%202015 per cent20Final%20version.pdf

[27] Maheswaran R , Elliott P, Strachan DP. Socioeconomic deprivation, ethnicity and stroke mortality in Greater London and south-east England. J Epidemiol Community Health 1997;51:127–131 9196639 10.1136/jech.51.2.127 PMC1060432

[28] Avendano M , Kawachi I, Van Lenthe F, Boshuizen H, Mackenbach J, Van den Bos G et al Socioeconomic status and stroke incidence in the US elderly: the role of risk factors in the EPESE study. Stroke 2006;37:1368–1373 16690902 10.1161/01.STR.0000221702.75002.66

[29] Grimaud O , Dufouil C, Alpérovitch A, Pico F, Ritchie K, Helmer C et al Incidence of ischaemic stroke according to income level among older people: the 3C study. Age Ageing 2011;40:116–121 21071453 10.1093/ageing/afq142 PMC3309883

[30] Maheswaran R , Tong T, Michaels J, Brindley P, Walters S, Nawaz S. Socioeconomic disparities in abdominal aortic aneurysm repair rates and survival. Br J Surg 2022;109:958–967 35950728 10.1093/bjs/znac222 PMC10364757

[31] Hart JT . The inverse care law. Lancet 1971;1:405–412 4100731 10.1016/s0140-6736(71)92410-x

[32] Walton E , Ahmed A, Burton C, Mathers N. Influences of socioeconomic deprivation on GPs’ decisions to refer patients to cardiology: a qualitative study. Br J Gen Pract 2018;68:e826–e834 30348887 10.3399/bjgp18X699785 PMC6255241

[33] Willems S , De Maesschalck S, Deveugele M, Derese A, De Maeseneer J. Socio-economic status of the patient and doctor-patient communication: does it make a difference? Patient Educ Couns 2005;56:139–146 15653242 10.1016/j.pec.2004.02.011

[34] Verlinde E , De Laender N, De Maesschalck S, Deveugele M, Willems S. The social gradient in doctor-patient communication. Int J Equity Health 2012;11:12 22409902 10.1186/1475-9276-11-12 PMC3317830

[35] Mercer SW , Zhou Y, Humphris GM, McConnachie A, Bakhshi A, Bikker A et al Multimorbidity and socioeconomic deprivation in primary care consultations. Ann Fam Med 2018;16:127–131 29531103 10.1370/afm.2202 PMC5847350

[36] FitzGerald C , Hurst S. Implicit bias in healthcare professionals: a systematic review. BMC Med Ethics 2017;18:19 28249596 10.1186/s12910-017-0179-8 PMC5333436

[37] Hisam B , Zogg CK, Chaudhary MA, Ahmed A, Khan H, Selvarajah S et al From understanding to action: interventions for surgical disparities. J Surg Res 2016;200:560–578 26526625 10.1016/j.jss.2015.09.016

