# Supplementary material for: Socioeconomic disparities in surgery for carotid artery disease in England
Source: BJS Open. 2023 Jul 28;7(4):zrad056. doi: 10.1093/bjsopen/zrad056 (PMC10375942; doi:10.1093/bjsopen/zrad056)
Supplement: zrad056_Supplementary_Data [file zrad056_supplementary_data.docx]

**Title:**

**Socioeconomic disparities in surgery for carotid artery disease in England**

**Authors:**

Thaison Tong ^1^

Ravi Maheswaran ^1^

Jonathan Michaels ^1^

Paul Brindley ^2^

Stephen Walters ^1^

Shah Nawaz ^3^

**Affiliations:**

^1^ School of Health and Related Research, University of Sheffield, United Kingdom

^2^ Department of Landscape Architecture, University of Sheffield, United Kingdom

^3^ Sheffield Vascular Institute, Sheffield Teaching Hospitals NHS Foundation Trust, United Kingdom

**Corresponding author:**

Professor Ravi Maheswaran, Public Health, School of Health and Related Research, Regent Court, 30 Regent Street, Sheffield S1 4DA, United Kingdom

Email: r.maheswaran@sheffield.ac.uk

ORCID ID: 0000-0002-3899-4421

**Supplementary Materials - Index**

**Supplementary Figures**

Figure S1 Page 2

Figure S2 Page 3

**Figure S1.** Annual time trends in population CEA rates by sex and age group; England (April 2006 – March 2018).


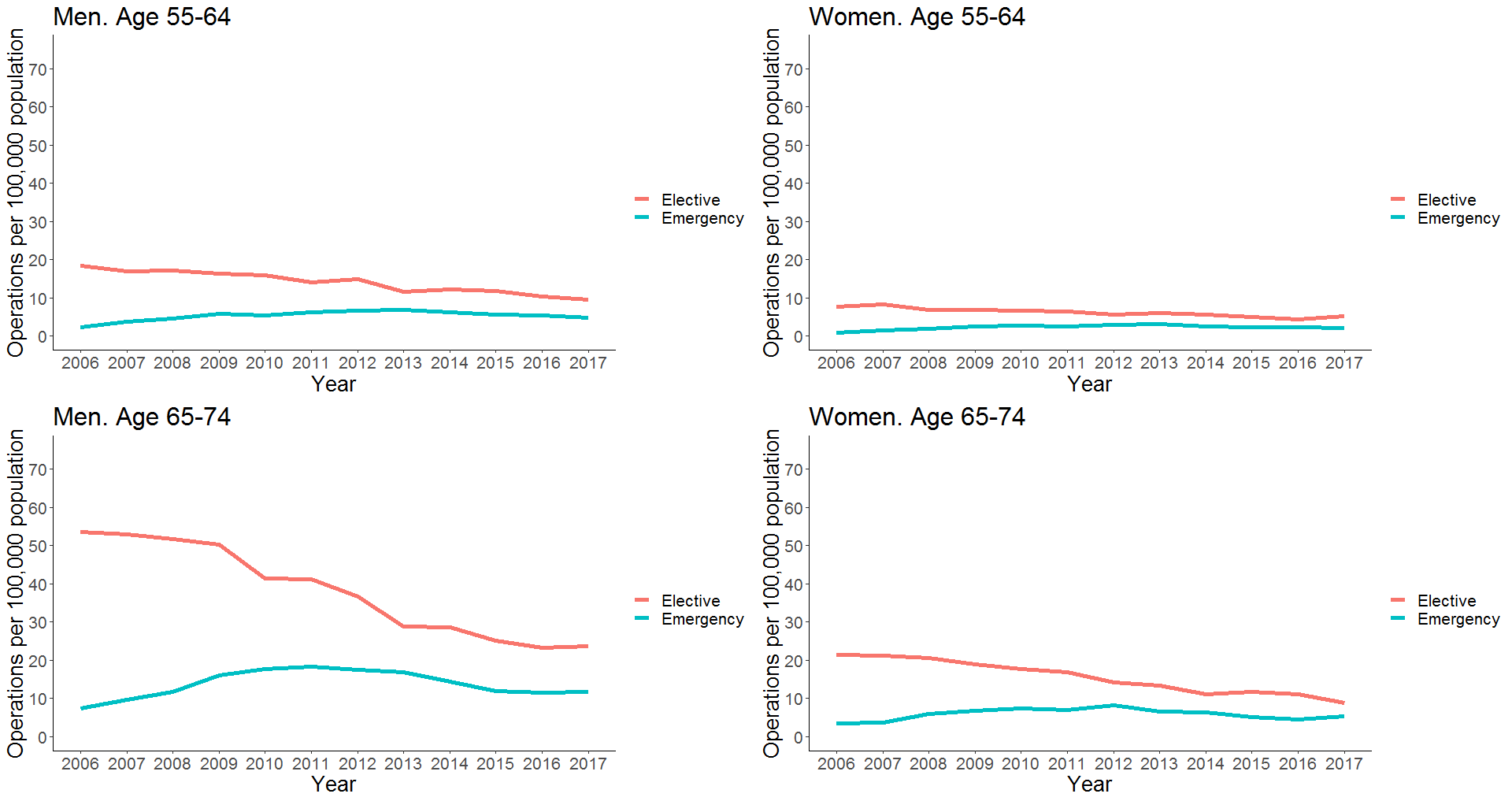


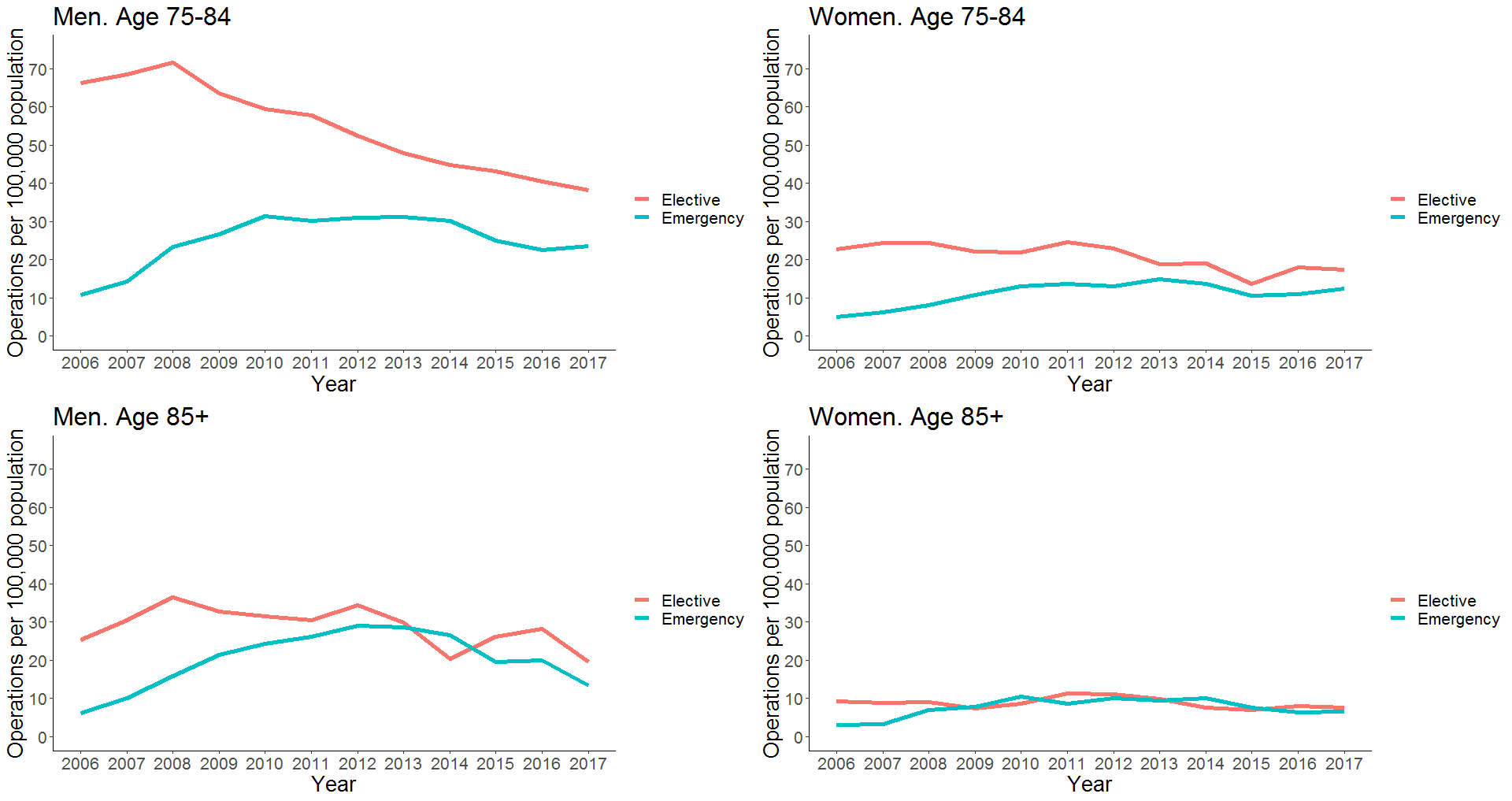


CEA – carotid endarterectomy

**Figure S2.** Survival following CAD repair by sex and type of procedure for all patients aged 55 years or more in England (April 2006 – March 2018).


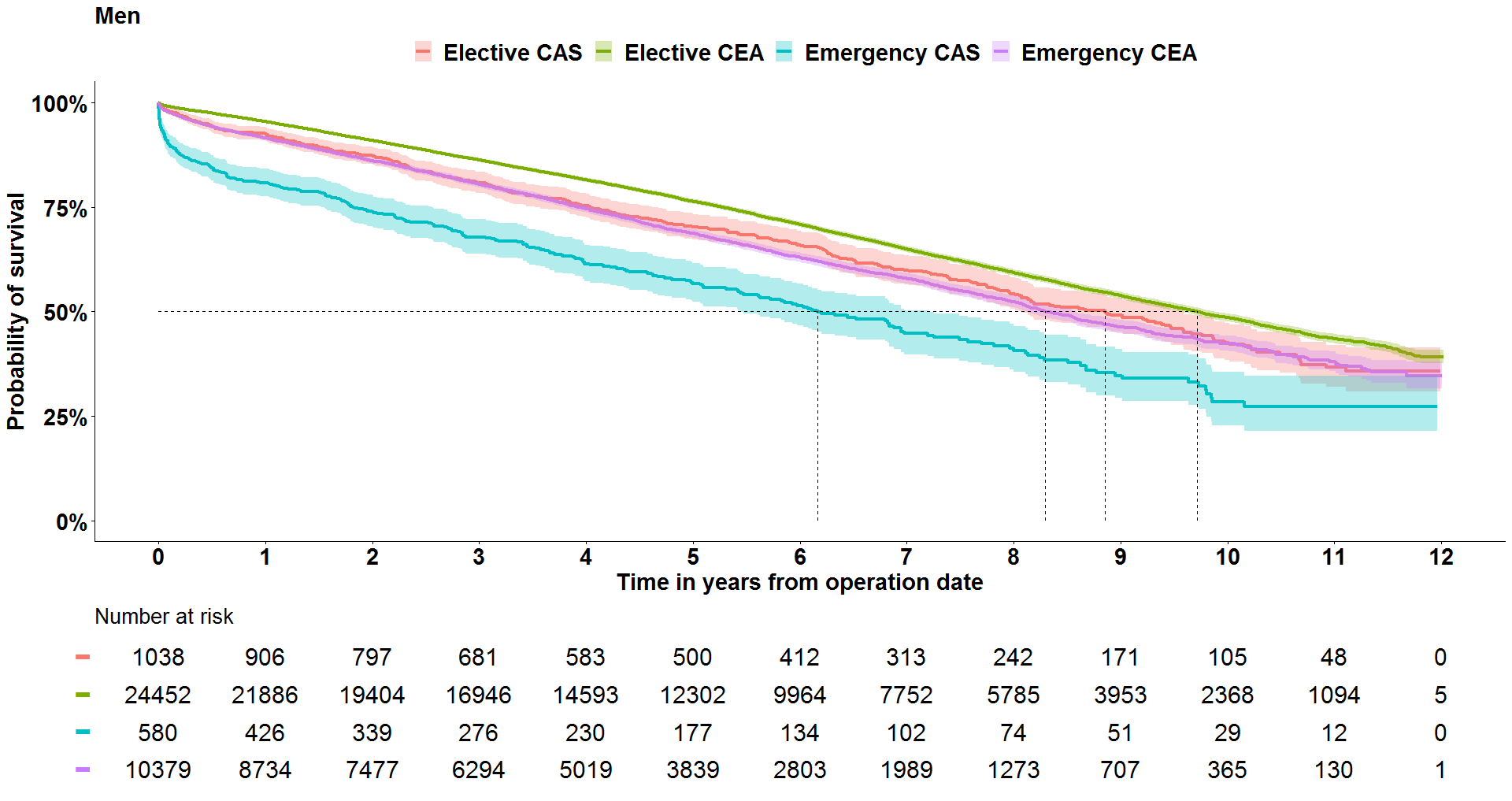


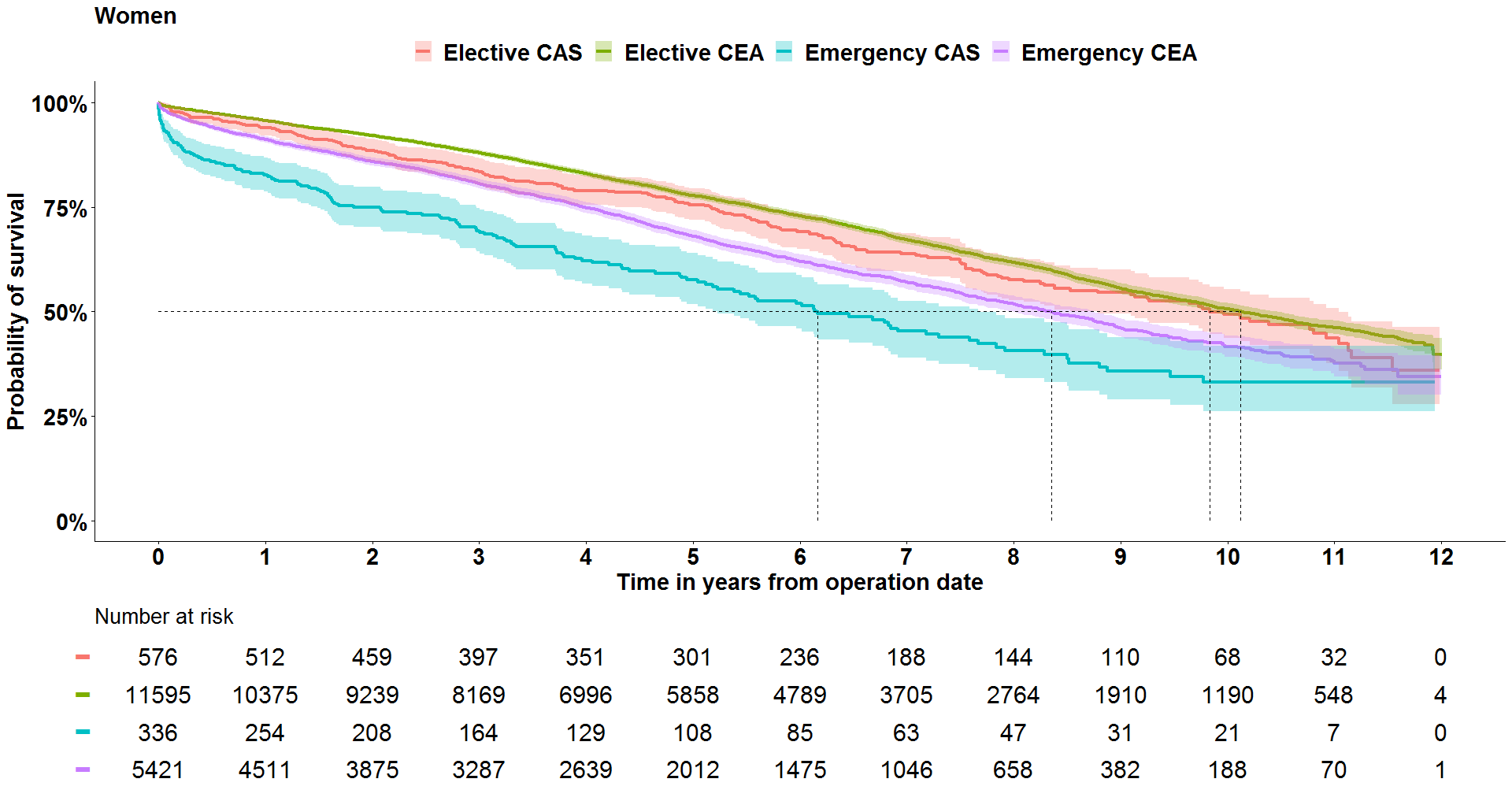


CAD – carotid artery disease; CEA – carotid endarterectomy; CAS – carotid artery stenting
